# Abnormal regional homogeneity and its relationship with symptom severity in cervical dystonia: a rest state fMRI study

**DOI:** 10.1186/s12883-021-02079-x

**Published:** 2021-02-05

**Authors:** Shubao Wei, Chunhui Lu, Xiuqiong Chen, Lu Yang, Jing Wei, Wenyan Jiang, Yang Liu, Hui Hui Li, Yuhong Qin, Yiwu Lei, Chao Qin, Caiyou Hu, Shuguang Luo

**Affiliations:** 1 Department of Rehabilitation Medicine, Jiangbin Hospital of Guangxi Zhuang Autonomous Region, Nanning, 530021 Guangxi China; 2 Department of Neurology, The First Affiliated Hospital of Guangxi Medical University, Nanning, 530021 Guangxi China; 3 Department of Radiology, the First Affiliated Hospital, Guangxi Medical University, Nanning, 530021 Guangxi China

**Keywords:** Cervical dystonia, Resting-state functional magnetic resonance, Regional homogeneity, Default mode network

## Abstract

**Background:**

Although several brain networks play important roles in cervical dystonia (CD) patients, regional homogeneity (ReHo) changes in CD patients have not been clarified. We investigated to explore ReHo in CD patients at rest and analyzed its correlations with symptom severity as measured by Tsui scale.

**Methods:**

A total of 19 CD patients and 21 gender-, age-, and education-matched healthy controls underwent fMRI scans at rest state. Data were analyzed by ReHo method.

**Results:**

Patients showed increased ReHo in the right cerebellum crus I and decreased ReHo in the right superior medial prefrontal cortex (MPFC). Moreover, the right precentral gyrus, right insula, and bilateral middle cingulate gyrus also showed increased ReHo values. A significantly positive correlation was observed between ReHo value in the right cerebellum crus I and symptom severity (*p* < 0.05).

**Conclusions:**

Our investigation suggested abnormal ReHo existed in brain regions of the “pain matrix” and salience network (the right insula and bilateral middle cingulate gyrus), the motor network (the right precentral gyrus), the cerebellum and MPFC and further highlighted the significance of these networks in the pathology of CD.

## Background

Cervical dystonia (CD) is a neurologic disorder characterized by involuntary sustained contractions of the cervical musculature, causing the head to rotate abnormally or tilt in a particular directions [1]. The head may typically turn to a specific direction resulting in torticollis, laterocollis, anterocollis or retrocollis [19]. CD is the most common form among the focal dystonia and frequently starting at later ages of 40–60 years. The disorder is frequently accompanied by head tremor and chronic neck pain [17]. Analysis of the influence of CD on work productivity has confirmed the substantial negative influence of CD on employment, with CD-related pain as a particularly important driver [27]. Moreover, CD patients sustain significantly psychosocial disability and decline of life quality. Thus, it is important to identify CD patients and provide them with effective treatment. However, the pathophysiology underlining the disorder is only partly understood.

Developments in neuroimaging techniques opened new avenues for detailed investigation of structural changes and regional activities in the brain involved in the pathophysiology of CD. Some CD patients show structural alterations in the basal gangli, thalamus, cerebellum, motor cortex, and supplementary motor cortices [9, 10, 29, 34, 35, 47]. In addition, a negative correlation between putamen volume and symptom severity in CD patients has been reported [8]. Functional magnetic resonance imaging (fMRI) results are consistent with that of structural neuroimaging. Results from fMRI researches demonstrate aberrant activation in basal ganglia, premotor, and motor-related areas [5, 6]. In general, growing evidences indicated that not only the basal ganglia but also the cerebellum and sensorimotor cortices may be conducive to the pathology of CD. However, results are not merely contradictory, and whether these changes are causative or compensatory is still uncertain. Therefore, the pathology of CD is remain unclear.

Independent component analysis (ICA) and seed-based region of interest (ROI) are two methods most widely employed for analyzing the resting-state data. Both methods present several significant benefits and disadvantages that have stated previously [44]. Given the shortcomings of both methods, the present study uses a method called regional homogeneity (ReHo) to examine the regional homogeneity in CD patients. In addition, asymmetric activity patterns have observed in CD patients [4, 6], but these patterns were remain uncertain. Thus, a measure of regional homogeneity might give a better insight on this aspect. ReHo is a measurement of similarity or synchronicity of the time series of nearest neighboring voxels. A lower ReHo may imply hypoactive in the regional area, and vice versa [46]. Aberrant ReHo could indicate the disturbance of temporal aspects of neural activity and be related to pathophysiology underlining disorder [37]. So far, ReHo has been well applied in study of schizophrenia, depression and somatization disorder [12, 13, 24, 37].

In order to investigate the regional homogeneity in CD patients, we used ReHo approach to analyze fMRI data at rest state. We hypothesize that CD patients would show abnormal regional homogeneity, particularly the motor-related areas.

## Methods

### Subjects

The study is conducted in an outpatient setting of the Department of Neurology, the First Affiliated Hospital, Guangxi Medical University, China. A total of 21 right-handed CD patients were originally recruited. CD was diagnosed by 2011EFNS (European Federation of Neurological Societies) guidelines on diagnosis and treatment of primary dystonias. Exclusion criteria for CD group were as follows: (1) secondary spasmodic torticollis that is definitely diagnosed; (2) any history of serious medical or neurological illness; (3) any history of botulinum toxin treatment, related medical treatment, or operation therapy in the three recent months; and (4) any history of neurological or psychiatric disorders.

Healthy controls were simultaneously recruited from the community. Each healthy control was right-handed and group-matched in gender, and age,. Exclusion criteria for the control group were as follows: (1) any history of serious medical or neurological illness; (2) any history of severe neuropsychiatric diseases; and (3) any family history of neurological or psychiatric disorders in their first-degree relatives. Participants who had any contraindications for MRI or shown changes under conventional MRI scans were excluded.

Every patients were evaluated using Tsui scale [43] to measure symptom severity of CD. All participants were showed the information related to the study procedures and signed the consent forms before evaluations. The ethics committee of the First Affiliated Hospital, Guangxi Medical University approved our study. All participants were given information regarding study procedures and subsequently provided written informed consent.

### Image acquisition

Resting-state scans were captured by using a Siemens 3.0 T scanner (Erlangen, Germany). Earplugs were applied for reducing scanner noise and foam padding for minimizing head movement. All participants were requested to lie still, stay awake with their eyes closed and relax. Each participant was asked to avoid thinking of anything in particular during the image acquisition. After the session, each subject was asked whether they had fallen asleep during the scanning. The one who responded positively or ambiguously was excluded. Echo-planar imaging sequence was acquired from each subject by using the following parameters: repetition time/echo time (TR/TE) = 2000/30 ms, slice thickness = 4 mm, gap = 0.4 mm, matrix = 64 × 64, flip angle = 90°, FOV = 24 cm × 24 cm, number of volumes = 250.

### Image pre-processing

The resting-state fMRI (DPARSF) [2] data processing assistant was used for image preprocessing in MATLAB [26]. We discarded the first ten images of each run to consider the equilibration of the signal. We first corrected for slice timing, and then conducted head motion. Afterward, participants with a maximum displacement of more than 2° motion and 2 mm in x, y, z (rotation and translation parameters) were excluded. Subsequently, fMRI images were normalized to the standard Montreal Neurological Institute (MNI) EPI space and resampling to 3 mm × 3 mm × 3 mm. Acquired images were temporally band-pass filtered (0.01–0.08 Hz) and linear detrended for the reduction of low-frequency drift and respiratory and cardiac noises.

### ReHo analyses

We performed ReHo analyses by MATLAB [26] (Mathworks) using software REST (http://www.resting-fmri.sourceforge.net). Kendall’s coefficient of concordance (KCC) was calculated to represent the ReHo of a specified voxel. In voxel-wise analysis, measurements of each subject’s ReHo were obtained by calculating the KCC of the time series of a particular voxel and its nearest neighbor (26 voxels). The calculation formula of KCC value have already expounded by Zang et al. [46]. For reducing the confounding of individual variations in KCC value, the normalization of ReHo maps was performed by dividing KCC among each voxel by the averaged KCC of the whole brain. Generated ReHo maps were then spatially smoothed with a Gaussian kernel of 8 mm full-width at half-maximum.

### Statistical analyses

Clinical and demographic information was calculated including age, sex between the patients and the control group and the average of illness duration, symptom severity in the patients group. Quantitative variables were compared by the two-sample *t*-test, whereas qualitative variables were compared by the χ2 test (*p* < 0.05). ReHo analyses were performed using REST through the two-sample *t*-tests. The level of significance was set at the corrected *p* < 0.005 using the Gaussian random field (GRF) correction method at the cluster level (voxel significance: *p* < 0.001, cluster significance: *p* < 0.005). We also respectively performed a voxel-based Pearson correlative analysis between mean ReHo values of clusters and the patients’ age, and Spearman correlative analysis between illness duration and Tsui total score. The level of significant correlation was set at *p* < 0.05.

## Results

### Subjects

There were no subject excluded due to any contraindications for MRI or shown changes under conventional MRI scans. No subjects were excluded due to falling asleep during image acquisition. Two patients with excessive head movement and excluded from further analysis. Consequently, a total of 19 patients and 21 healthy controls were included in further analysis. There were no significant differences in age, and sex ratio between the patient group and the control group. Information on the demographic and clinical characteristics of included subjects is detailedly given in Table 1. In addition, a total of 17 patients reported neck pain; 7 patients were left-side affected and the others were bilateral-side affected; a total of 18 of the 19 patients reported sensory tricks, that is the abnormal posture and involuntary movement of the head and neck can be temporarily improved from light touching the lower part of the cheek, jaw and posterior neck and leaning against the wall, as well as keeping something in the mouth and carrying a weight backpack on the back. Only one patient has never reported sensory tricks.

**Table 1:** Demographics and clinical characteristics of the patients and the controls

Variables (mean ± standard deviation)	Patients	Controls	*p* value
Gender (female/male)	10/9	15/6	0.220^a^
Age, years	38.74 ± 10.71	39.62 ± 6.62	0.759^b^
Illness duration, months	24.29 ± 31.26		
Tsui	16.32 ± 4.45		

Tsui: Tsui scale

^a^The *p* value for gender distribution in the two groups was obtained by chi-square test

^b^The *p* values were obtained by two sample *t*-tests

### ReHo: between-group comparison

**Fig. 1:**
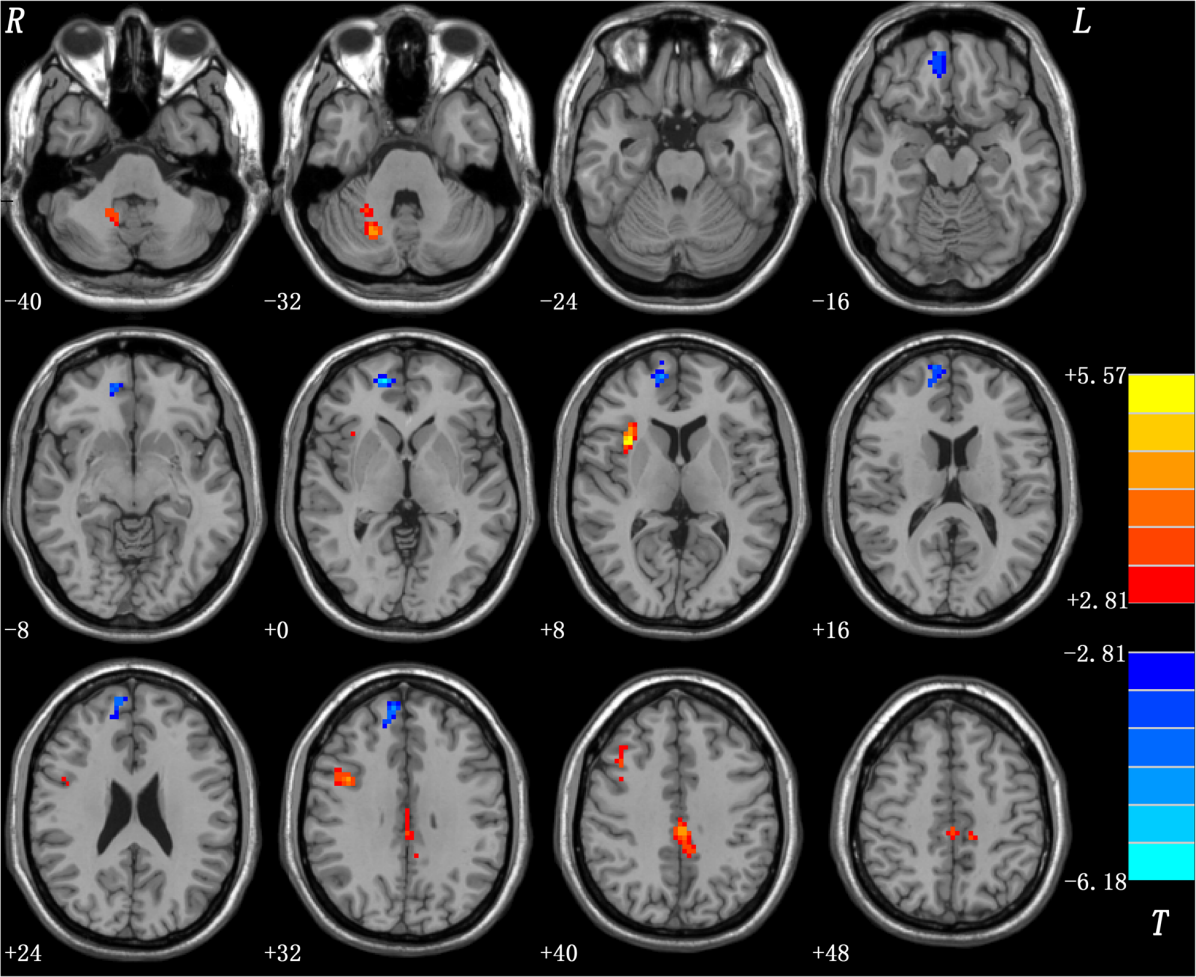
ReHo differences between patients with CD and controls. Red and blue denote higher and lower ReHo respectively and the color bars represent the *t* values from two-sample *t*-test of the group analysis. ReHo = regional homogeneity; CD = cervical dystonia

Significant differences between ReHo values of the patient and control groups were observed within the whole brain based on the two-sample *t*-tests by voxelwise cross-subject comparisons. Compared with healthy controls, CD patients had higher ReHo in the right cerebellum crus I, right insula, right precentral gyrus, and bilateral middle cingulate gyrus but lower ReHo in the right superior medial prefrontal cortex (MPFC) (Fig. 1 and Table 2).

**Table 2:** Brain regions with significant ReHo differences in the patients

Brain regions	Peak (MNI)			Number of voxels	*T* value
	x	y	z		
*Patients > Controls*					
Right Cerebellum Crus I	24	−66	−33	54	4.5063
Right Insula	33	15	9	46	5.5657
Right Precentral Gyrus	39	6	33	68	4.4075
Bilateral Middle Cingulate Gyrus	0	−27	42	125	5.0219
*Patients < Controls*					
Right Superior MPFC	15	54	3	193	−6.1797

x, y, z, coordinates of primary peak locations in the MNI space; *T* statistical value of peak voxel showing ReHo differences between the patients with CD and the controls; CD: cervical dystonia; *ReHo* regional homogeneity, *MNI* Montreal Neurological Institute, *MPFC* medial prefrontal cortex

### Correlations between ReHo and clinical variables

**Fig. 2:**
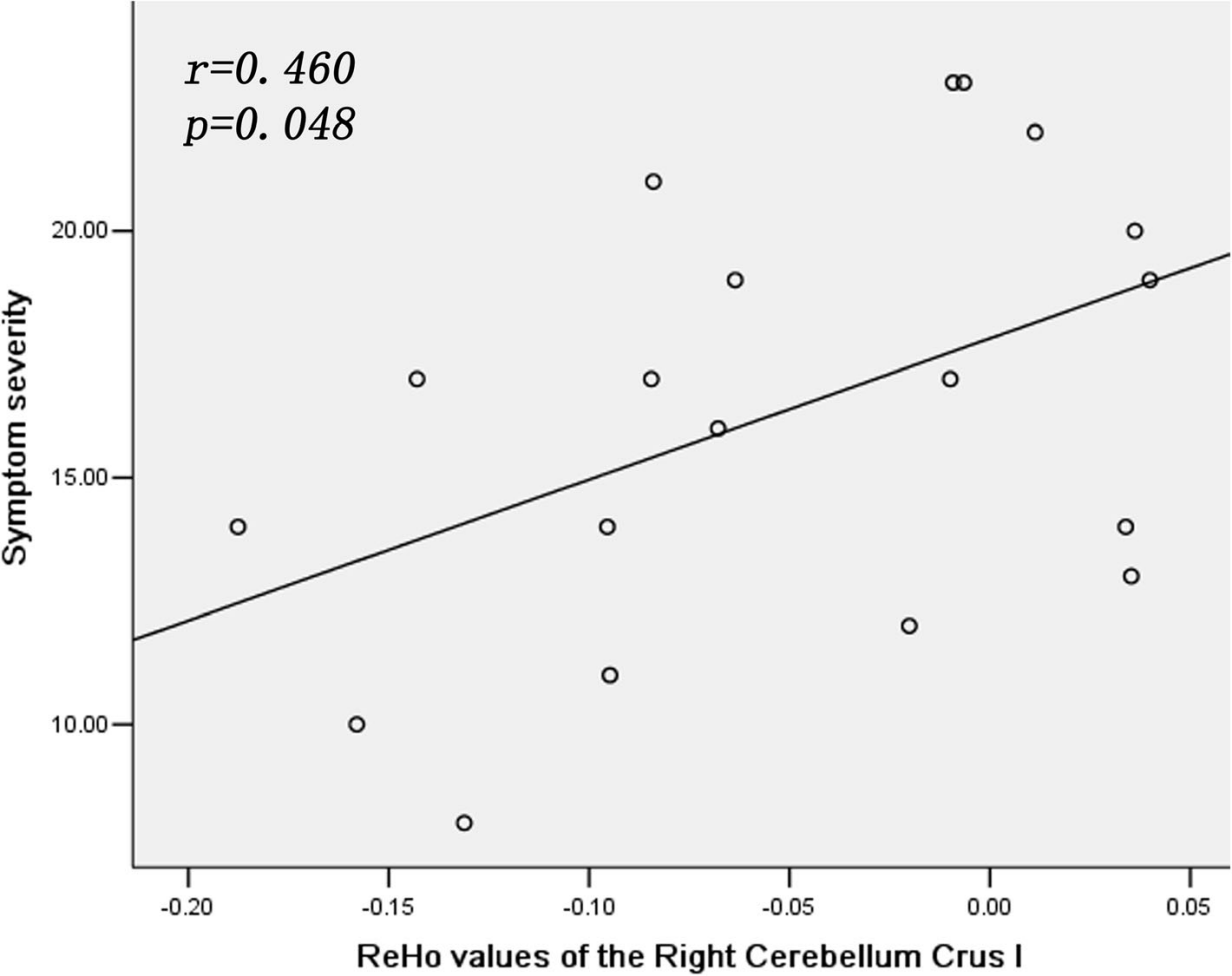
Positive correlation between ReHo in Right Cerebellum Crus I and symptom severity in the patient group. ReHo = regional homogeneity. The correlation between ReHo in Right Cerebellum Crus I and symptom severity was obtained by Pearson correlation analysis

A significant positive correlation was observed between ReHo value in the right cerebellum crus I and symptom severity at *p* < 0.05 (Fig. 2). Linear correlations in the patient group were evaluated between ReHo and illness duration, and age; no significant correlations were detected, *p* > 0.05.

## Discussion

ReHo is a measure to detect the similarity or synchronization of the time series of nearest neighboring voxels (usually 27 voxels) with the calculation of KCC. This approach was used with fMRI data at rest to investigate the regional homogeneity in CD. Compared with controls, CD patients showed higher ReHo in the right insula and the bilateral middle cingulate gyrus, the right precentral gyrus, and the right cerebellum crus I but lower ReHo in the right superior MPFC.. Furthermore, a significant positive correlation was observed between the ReHo value in the right cerebellum crus I and the symptom severity.

By usage, the dystonia that covered the cervical dystonia has been attributed to the dysfunction of the basal ganglia. However, the cerebellum has been recently suggested as a probable originate region. Traditionally, the cerebellum is identified as a region engaged in motor coordination. It plays a pivotal role in modulation of premotor, sensorimotor and posterior parietal regions for fine-tuning motor control. Pong et al. have demonstrated that the output of the cerebellum and the output of from the basal ganglia work together to participate in movement control of the head and face [33]. Moreover, the cerebellum has been suggested as a processor of sensory information, integrating descending visual input from the parietal cortex and ascending input from the spinocerebellar pathway for promoting a forward model, as well as for predicting sensory consequences of an action [45]. Given these findings, we have reason to believe that the abnormality in the right cerebellum crus I with CD could influenced the accuration of the movement control of the head and face impaired because the abnormal modulation.

It is noteworthy that a significant positive correlation was observed between ReHo value in the right cerebellum crus I and symptom severity in the present study. Previously, an animal studies observed that pharmacological exciting of the cerebellum leads to dystonia [32]. These findings supported the conjecture that dystonia is caused by aberrant, distorted functional output [20]. Based on these notions, the result of a higher ReHo value in the right cerebellum crus I in CD patients here consistently indicated that the dysfunction of this region can be a key factor in the occurrence of motor symptoms in CD, which highlighted the importance of the cerebellum for motor modulation in the pathology of cervical dystonia.

Anatomically, MPFC composed of discrete and cytoarchitectonically areas receiving large-scale of sensory information from the external environment and the body [39]. Specifically, the lobule Crus I of the cerebellum closely associated with MPFC [28], agrees with a prominent role of both regions in non-motor functions [21]. Functionally, MPFC was proposed to be involved in many higher executive functions, involving in emotion, decision-making, goal-directed behavior, working memory and attention [25]. Therefore, we proposed that the decreased ReHo value of the right superior MPFC possibly influence this region’s function and result in losing top-down regulation, which is suggested as the foundation in the pathology of changes of cognitive, emotional processing, and behavior in CD. In our other study, we have found patients with CD exhibit significantly decreased VMHC in superior MPFC [18]. Besides, changes in the cognitive processing of movement have been previously observed in idiopathic dystonia patients [15, 23]. In other studies, in addition to organization and execution of movement, aberrant motor cognition consisting of a mental rotation of body parts, temporal processing and alterations of movement, and body representation have been observed in CD [7]. These findings strengthen our conjecture.

Additionally, in animal study, MPFC was confirmed receiving afferent projections representing all sensory modalities, which originated from widespread areas of the cortex (and associated thalamic nuclei). The dorsal MPFC presumably integrated and utilized this information for goal directed actions [16]. Thus, abnormal neuro-activity in MPFC may influence goal directed actions. On this basis, the “sensory trick”, which we suggested as a goal directed action, may have a correlation with the changes of MPFC. In the present study, the sensory trick phenomenon was observed in 18 patients, which was consistent with previous report [11]. These patients usually acquire attenuation of abnormal head movements by slightly touching a particular area of the face or head. It has previously been proposed to use sensory techniques to influence proprioceptive input to balance the inhibition ratio to facilitation [36]. Recently, the sensory trick was suggested as a modulation of abnormal connections between sensory input and motor output [11]. The inconsistent standpoint renders the mechanism latent the sensory trick phenomenon still intricate. Our findings probably supported the latter. Consequently, we suggested MPFC involved in the mechanism of the sensory trick.

Increasing evidence emphasizes dystonia as a disorder of motor organization, programming, sensorimotor, and execution [7]. the primary motor cortex (as the M1), that is the precentral gyrus, received projections from BA2 and BA5 areas that contain the contralateral cutaneous, muscular and articular information, and subsequently corrected movement. Meanwhile, as the target of projections from frontal cortical regions and subcortical regions, precentral gyrus is a probable site converging mechanisms of the selection, initiation and inhibition movement [38]. This connectional architecture induced the precentral gyrus to play a crucial role in the mechanism of generated CD. The abnormality in motor system physiology of patients with dystonia was exhibited by reduced surround inhibition resulting in unnecessary contractions of more muscles than what is required for specified motor behavior [5]. Therefore, the increased ReHo in the right precentral gyrus in CD patients could lead to impaired selection, initiation or inhibition of movement though impairing the cortices - basal ganglia – cortices circuit. In other words, it was in line with the notion that the cervical dystonia has been attributed to not only the dysfunction of the basal ganglia but also the cortices. In our other study, patients with CD showed abnormal activities with decreasing GFC in the the M1-SMA motor network, including right supplementary motor area and right precentral gyrus. Moreover, the GFC values in the right precentral gyrus of CD patients was significantly negative correlated to the symptomatic severity [30]. Hence, abnormal regional homogeneity in this region is important for the pathology of CD.

Pain is the most common and disabling non-motor symptom and contributes significantly to patient disability and low quality of life in CD. The incidence of pain is reported up to 88.9% and most of them rated their pain as moderate or severe [3]. In the present study, a total of 17 patients (89.4%) reported painful neck muscles. The ratio was close to that reported in previous study. So far, causative factors of CD-related pain are still a matter of debate whether excessive muscle contractions or alterations of transmission and processing of nociceptive stimuli [41]. Study has released cutaneous nociceptive pathway function in CD patients is normal [42]. This generates a hypothesis of the crucial note of CD-related pain may correlate to the centre nervous system.

It is worthy to note that the right insula and the bilateral middle cingulate gyrus exhibited an increased ReHo in CD patients. Both two regions have been confirmed to be related to pain perception [31], so that they were recognized as brain regions belonging to the “pain matrix”. In masses of previous findings, CD was linked to multiple brain regions but rarely to the insula and the cingulate gyrus. In fact, it is well known that the different brain areas constituting the “pain matrix”, including SII, the insula and the anterior cingulate cortex. The same areas are part of the salience network circuit which is linked to pain [22]. Moreover, the insular and cingulate cortex play crucial roles in integrating multimodal information significant for several functions, such as sensorimotor, allostatic/homeostatic, emotional, and cognitive functions [40]. On these basis, the abnormal ReHo values in these brain areas might be of important significance. The increased ReHo in the right insula and the bilateral middle cingulate gyrus may influenced the functions of the “pain matrix” and/or the salience network circuit consequently resulted the generation or impair processing of CD-related pain and induce an abnormal sensorimotor function in CD patients. Based on our results and the reasoning aforementioned, we speculated that CD-related might caused by alterations of transmission and processing of nociceptive stimuli. Thus, the observation of abnormal activities in these brain areas extended the understandings of the pathology underlying CD.

We observed asymmetric activity patterns in CD that were strongly involved in the right-hemispheric dystonia-related connectivity pattern. This phenomenon has been previously reported, for instance, during finger movements [4], and at resting-state [6]. It was possibly associated to the larger number of contralateral-side muscles affected patients. However, patients in our study were primary bilateral-side muscles affected. A possible explanation for the laterality of the right-hemispheric may be due to its dominance of position control, that is the right hemisphere determine response modification so that it is dominant for position control [14].

Aside from the small sample size, several limitations must be stated in this study. First, all of the patients’ dystonic posturing was minimal in the supine position or absent during scanning. To confirm whether this is a specific sensory trick is difficult. Therefore, influences of sensory trick could not be easily eliminated. Besides, physiological noises including heart rhythm and respiratory cannot be completely eliminated even though a relatively low sampling rate (TR = 2 s) is used. Lastly, with a small sample size, patients were not subdivided further into different groups according to head rotation. Hence, research using a larger sample size is required to expand our results.

## Conclusions

Our investigation suggested abnormal ReHo existed in brain regions of the “pain matrix” and salience network (the right insula and bilateral middle cingulate gyrus), the motor network (the right precentral gyrus), the cerebellum and MPFC and further highlighted the significance of these networks in the pathology of CD.

## Data Availability

The datasets used and analyzed during the current study available from the corresponding author on reasonable request.
